# TDDFusion: A Target-Driven Dual Branch Network for Infrared and Visible Image Fusion

**DOI:** 10.3390/s24010020

**Published:** 2023-12-19

**Authors:** Siyu Lu, Xiangzhou Ye, Junmin Rao, Fanming Li, Shijian Liu

**Affiliations:** 1 School of Information Science and Technology, ShanghaiTech University, Shanghai 201210, China; lusy@shanghaitech.edu.cn; 2 Shanghai Institute of Technical Physics, Chinese Academy of Sciences, Shanghai 200083, China; yexiangzhou@mail.sitp.ac.cn (X.Y.); raojunmin@mail.sitp.ac.cn (J.R.); lifanming@mail.sitp.ac.cn (F.L.); 3 Key Laboratory of Infrared System Detection and Imaging Technology, Chinese Academy of Sciences, Shanghai 200083, China; 4 University of Chinese Academy of Sciences, Beijing 100049, China

**Keywords:** image fusion, target-driven, high-level vision task, vision transformer, deep learning

## Abstract

In the field of image fusion, the integration of infrared and visible images aims to combine complementary features into a unified representation. However, not all regions within an image bear equal importance. Target objects, often pivotal in subsequent decision-making processes, warrant particular attention. Conventional deep-learning approaches for image fusion primarily focus on optimizing textural detail across the entire image at a pixel level, neglecting the pivotal role of target objects and their relevance to downstream visual tasks. In response to these limitations, TDDFusion, a Target-Driven Dual-Branch Fusion Network, has been introduced. It is explicitly designed to enhance the prominence of target objects within the fused image, thereby bridging the existing performance disparity between pixel-level fusion and downstream object detection tasks. The architecture consists of a parallel, dual-branch feature extraction network, incorporating a Global Semantic Transformer (GST) and a Local Texture Encoder (LTE). During the training phase, a dedicated object detection submodule is integrated to backpropagate semantic loss into the fusion network, enabling task-oriented optimization of the fusion process. A novel loss function is devised, leveraging target positional information to amplify visual contrast and detail specific to target objects. Extensive experimental evaluation on three public datasets demonstrates the model’s superiority in preserving global environmental information and local detail, outperforming state-of-the-art alternatives in balancing pixel intensity and maintaining the texture of target objects. Most importantly, it exhibits significant advantages in downstream object detection tasks.

## 1. Introduction

With the rapid development of sensing technologies, multimodal image fusion has emerged as a pivotal technique in the realms of image processing and computer vision. In particular, the fusion of infrared and visible light images holds great potential for enhancing system performance and robustness. Infrared imaging captures thermal radiation from objects, which is especially useful in low-light conditions, while visible light imaging provides high-resolution texture details. However, infrared images typically lack texture information, and visible light imaging is highly sensitive to lighting conditions, limiting their effectiveness when used alone. The fusion of infrared and visible light overcomes these limitations by integrating their respective strengths. Such fused images not only highlight thermal targets but also enrich texture details, adapting well to lighting changes. This significantly improves the execution of advanced visual tasks, such as object detection [1,2], tracking [3,4], and segmentation  [5]. This technological advancement is crucial for improving the autonomous decision-making abilities of machines in complex environments, particularly in critical areas such as military surveillance [6], security monitoring [7], and autonomous driving [8]. Therefore, exploring effective multimodal fusion algorithms is of significant practical importance for accomplishing advanced visual tasks.

With the substantial value of infrared and visible light image fusion across various applications, there has been significant scholarly interest. The rise of deep learning has spurred numerous fusion methods, including those based on Convolutional Neural Networks [9,10,11,12], Autoencoders [13,14,15,16], and Generative Adversarial Networks [17,18,19,20]. While these methods have achieved commendable results, several challenges remain. On the one hand, most fusion techniques disconnect the relation with subsequent detection tasks, relying solely on visual effects and quantitative evaluations as the basis for fusion. In reality, subsequent detection is key to many practical computer vision applications. On the other hand, most existing methods for infrared and visible light image fusion apply a global and uniform process to the fused image. However, the fusion effect in the target area is more noteworthy and holds greater significance for downstream detection tasks. Some recent studies have focused on the impact of image fusion on downstream tasks [5,21], but most lack attention to the target itself. A few studies [22,23,24] have combined the SOD (Salient Object Detection) task with image fusion, focusing on target areas through salient object masks. Yet, saliency detection yields a singular foreground target, making it more suitable for uncluttered single-object scenarios. Consequently, it is less effective in handling the complexities of real-world road scenes. Moreover, these studies assume that target information comes from infrared images and detail information from visible light images. This assumption often leads to the loss of texture detail in visible light images, reducing targets to mere brightness. Such a reduction can obscure the target’s visual details, potentially diminishing the effectiveness of subsequent detection tasks.

To address these challenges, this paper proposes a joint network framework that synergizes image fusion and object detection tasks. Feedback from the detection module is backpropagated to the fusion module, thereby refining the image fusion process to better align with the requirements of the detection task. To effectively capture global information and enhance downstream detection tasks, a dual-branch feature extraction network is introduced, consisting of a Global Semantic Extractor (GST) and a Local Texture Encoder (LTE). The GST analyzes high-level semantic information of images from various spectrums using the Vision Transformer structure, while the LTE uses Convolutional Neural Networks combined with gradient residual dense blocks for precise local feature capture. This approach yields fused images that are visually appealing and exhibit superior performance in object detection tasks.

To sum up, the main contributions of this work can be summarized as follows:

- A detection-driven fusion framework for infrared and visible light images is proposed. By backpropagating feedback from the detection module to the fusion module, this framework efficiently achieves exceptional performance in both image fusion and downstream detection tasks.
- A novel objective loss function for the fusion task is introduced, designed to balance intensity and detail features in target regions. This effectively enhances fusion quality in these areas.
- A dual-branch feature extraction network is designed, incorporating Global Semantic Extractor (GST) and Local Texture Encoder (LTE) modules to handle global and local features, respectively. This design effectively enhances the model’s ability to extract both semantic and detailed information.
- Comprehensive experiments on public image fusion datasets, including LLVIP [25], TNO [26], and RoadScene [27], indicate that the proposed approach surpasses current general fusion frameworks and notably improves the performance of downstream detection tasks.

## 2. Related Work

### 2.1. AE and GAN Based Fusion Network

Recent studies have leveraged deep learning techniques, especially Convolutional Neural Networks (CNNs) and Autoencoders (AEs), to enhance the quality and effectiveness of image fusion. AE and CNN-based methods adapt conventional workflows from traditional image fusion algorithms for feature extraction, fusion, and reconstruction. These algorithms train auto-encoders to perform both feature extraction and reconstruction tasks. Li et al. [28] proposed an encoding–decoding framework in 2018. Within the encoder, each convolutional layer’s input is designed to be the output of all preceding convolutional layers. This dense connectivity serves for feature extraction, while the decoder is responsible for feature reconstruction and image fusion. To exploit deep features more effectively, Li et al. introduced NestFuse [29], which utilizes a multi-scale encoder and a nested-connected decoder. It fuses multi-scale features at each scale through a spatial and channel attention model. However, this approach still relies on manually designed fusion rules and discards the fusion strategy during training, limiting performance improvement.

To address these challenges, subsequent research shifted towards adopting a two-stage, end-to-end model structure. Studies in references [30,31] utilized a two-stage training strategy. They trained the fusion model separately in the second stage, simplifying the simultaneous tasks of feature extraction and fusion scheme determination. In the realm of end-to-end fusion models, both IFCNN [32] and U2Fusion [27] employed a general image fusion framework grounded in Convolutional Neural Networks. IFCNN adopts a straightforward and general network structure, while U2Fusion integrates a continuous learning approach, broadening its applicability across multiple fusion tasks. Ref. [33] introduced a dual-channel multi-scale fusion model guided by a thermal target mask. This mask, derived by suppressing the infrared image’s background, accentuates thermal objects, reducing cross-modal interference. However, the mask generation technique might pose challenges in complex scenes and retaining comprehensive semantic information. Lastly, PIAFusion [34], is a progressive image fusion network founded on illumination perception. It determines day and night based on overarching illumination perception to guide the fusion process, which could potentially overlook specific conditions of individual target objects.

Fusion methodologies based on Generative Adversarial Networks (GANs) leverage the adversarial training paradigm. In this framework, the generator synthesizes fused images while the discriminator assesses their quality, facilitating superior fusion outcomes. Ma et al. [19] were pioneers in applying GAN-based networks to the task of fusing infrared and visible images, thereby showcasing the considerable utility of adversarial training in this specific fusion domain. AWFGAN [35] introduced a fusion network for infrared and visible light images, employing attention guidance and wavelet constraints. An infrared mask is utilized to direct focus toward target regions in the infrared imagery. Though GAN-based fusion maintains good information quality and visual appeal, the unpredictability of adversarial training can lead to blurred edges in the fused images.

### 2.2. Vision Transformer

In the realm of infrared and visible light image fusion, early works predominantly leaned on Convolutional Neural Networks (CNN) and their derivatives for feature extraction. While CNNs excel in feature extraction and generalization capabilities, they underperform in capturing long-range dependencies within images [36]. Consequently, the inadequacy of global features further hampers fusion quality. Concurrently, ever since the Transformer model was introduced by Vaswani et al. in 2017 [37], it has achieved remarkable success in the Natural Language Processing (NLP) domain. The inception of ViT entailed a successful foray of transformers into computer vision tasks [38]. Notably, the integration of multi-head self-attention mechanisms addressed the shortcomings of CNNs in capturing long-range dependencies. Transformers have not only showcased stellar performance in text but also in visual tasks such as image classification, segmentation, and object detection [39,40,41].

Recently, transformers have been extensively applied and researched for image fusion tasks. For instance, the SwinFuse model [42] deploys full self-attention for image fusion, although it has some limitations in handling fine-grained information. On the other hand, Tang et al. proposed a dual-branch network utilizing transformers to extract globally salient information. This approach enables the model to consider the content of the image more comprehensively [43]. Similarly, Zhao et al.’s CDDfuse [44] decomposes images into base and detail layers, where the transformer block leverages distant attention to handle low-frequency global features.

Although the Transformer has demonstrated its effectiveness in image fusion tasks by adeptly handling global semantic information, the fusion model still confronts two inherent constraints. Firstly, in the absence of explicit semantic guidance, the model might fail to capture critical information accurately. Secondly, the fusion task inherently lacks ground truth as an optimization objective. Relying solely on pixel-level discrepancies might not be sufficient to unleash the full potential of the Transformer.

### 2.3. The High-Level Vision Tasks

Primary visual processing often serves as a preprocessing step for subsequent complex visual tasks. Hence, developing an elementary visual algorithm guided by advanced visual tasks can enhance the performance of later intricate visual processes. In 2017, AOD-Net by Li et al. [45] pioneered the study of the interplay between dehazing algorithms and high-level visual tasks. This streamlined dehazing network offers an integrated solution for advanced visual processing of degraded images. Guo et al. [46] introduced a semantic segmentation-driven deraining model tailored for autonomous driving, ensuring both rain removal and preservation of semantic details, thereby optimizing downstream semantic segmentation.

The intersection of image fusion and high-level visual tasks in the infrared and visible light spectrum has garnered recent attention. Hou et al. [20] developed SSGAN, an image fusion model leveraging Generative Adversarial Networks, which segregates source imagery into semantic foreground and background. In 2022, Tang et al. [5] proposed SeAFusion, which cascades the image fusion module with the semantic segmentation module. By utilizing semantic loss, they effectively directed the high-level semantic information flow back to the image fusion module, substantially enhancing the performance of advanced visual tasks on the fused images. While the use of the segmentation module to backpropagate semantic information indeed amplifies the semantic information in fused images, promoting downstream tasks, the model still employs a global uniform approach, failing to emphasize the prominence of target objects. Some researchers have also begun to integrate SOD tasks with image fusion. Ma et al.’s STDFusionNet [23] integrates salient object detection into infrared and visible light fusion, ensuring spatial coherence and the prominence of infrared features. In 2023, Wang et al. [47] explored the symbiosis between fusion and salient object detection in infrared and visible imagery, utilizing a feature-filtering fusion subnetwork and subsequent SOD processing. TarDAL [48] represents a cohesive approach to fusion and detection, extracting salient foregrounds and synthesizing modality insights, emphasizing infrared structure and visible texture preservation.

These studies have significantly delved into enhancing the visual perceptual quality and semantic information of crucial targets within fused images. Nevertheless, there remain imperative issues awaiting refinement. For instance, saliency detection predominantly captures singular foreground objects, which is apt for sparse single-object scenarios but falters in handling intricate multi-object road scenes. Furthermore, an inherent assumption in these methodologies posits that target information is primarily derived from infrared images while details emerge from visible light images. This assumption, often misaligned with real-world scenarios, might lead to a loss of inherent texture information from visible light, inadvertently diminishing the visual perception of the target in certain contexts, which could detrimentally impact subsequent tasks.

To address these problems, a joint network framework is introduced that synergistically enhances both image fusion and object detection tasks. Feedback from the detection module is backpropagated into the fusion process to guide the image fusion task. Additionally, by leveraging the positional information of the target acquired from the detection module, the visual prominence of the objects of interest is amplified during fusion. This approach not only produces fused images with increased visual appeal but also excels in object detection performance.

## 3. Proposed Method

This section introduces the structure and the loss function of the proposed target-driven fusion framework.

### 3.1. General Framework

In practical applications, image fusion technology serves as an invaluable tool for the integration of critical information from multiple imaging sources. Within the realm of infrared and visible light image fusion, conventional methods frequently overlook the facilitative role that image fusion plays in subsequent high-level vision tasks, such as object detection. In light of these limitations, this study introduces an innovative image fusion framework that not only emphasizes the complementary information between infrared and visible images but also refines the visual representation of target objects. Furthermore, this framework enhances the accuracy of downstream object detection tasks by incorporating backpropagation information from a target detection model.

**Figure 1:**
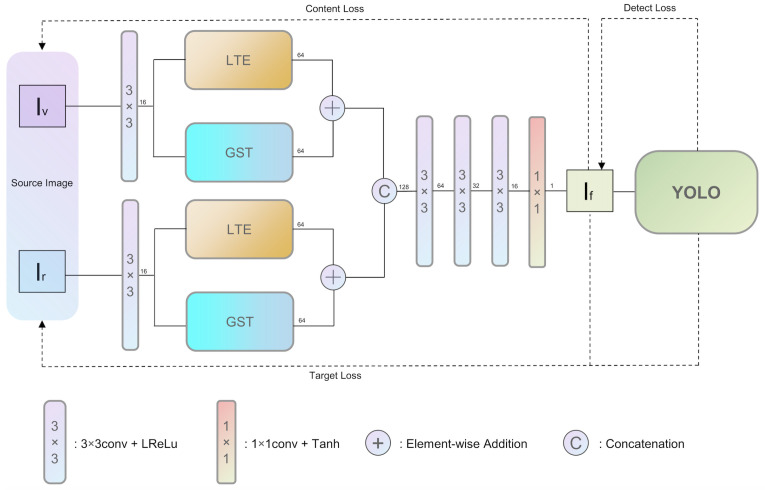
Overview of the TDDFusion model architecture.

**Figure 2:**
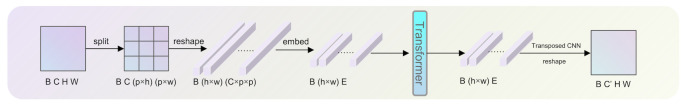
The framework of spatial transformer in GST module.

**Figure 3:**
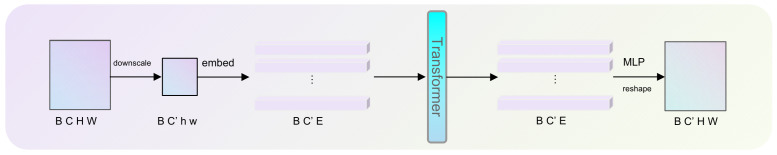
The framework of channel transformer in GST module.

**Figure 4:**
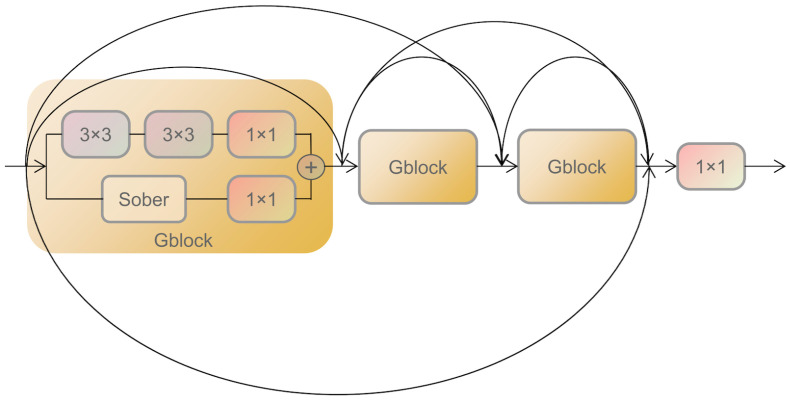
Architecture of the proposed LTE module.

The overall framework of the proposed network is depicted in Figure 1. The model employs a dual-branch design, featuring a GST module based on a Transformer architecture, and a LTE module implemented via Convolutional Neural Networks. As illustrated in Figure 2 and Figure 3, the GST leverages a Vision Transformer structure to profoundly capture high-level semantic information from multi-spectral images. Concurrently, the LTE utilizes Convolutional Neural Networks and Gradient Residual Dense Blocks to precisely capture local textural features, as demonstrated in Figure 4. These dual branches operate concurrently and their feature representations are integrated via a specialized decoding mechanism. To enhance the saliency and visual discriminability of target objects in the resultant fused imagery and improve the efficacy of downstream object detection tasks, a YOLO framework is cascaded post-fusion. Additionally, target-oriented loss functions, including detection loss and target loss, are introduced.

Moreover, inspired by the SeaFusion [5], two-stage iterative training strategy is adopted to maintain a balanced performance between the fusion and detection tasks. The specific algorithmic flow is detailed in Algorithm 1, with the number of iterations set to M. In the first stage, training of the fusion module is carried out while the parameters of the detection module remain frozen, allowing for backpropagation of detection loss to refine the fusion model. Exceptionally, when M = 0, which is the first round of iteration, the fusion model is trained solely based on content loss, as the detection module has not yet been fine-tuned for the fused images. In the second stage, training of the detection module is conducted based on the fused images outputted from the first stage. This two-stage process is iteratively conducted M times to achieve comprehensive training of the entire model.

### 3.2. Loss Function

In fusion tasks where a true ground truth is absent, the design of the loss function becomes crucial. We introduce a composite fusion loss $L_{\mathrm{fusion}}$ to guide the network. The loss is defined as follows:

(1) $$L_{\mathrm{fusion}}=\alpha L_{\mathrm{con}}+\beta L_{\det}+\gamma L_{\mathrm{tar}}$$

where $L_{\mathrm{con}}$ denotes the content loss, $L_{\det}$ signifies detection loss, and $L_{\mathrm{target}}$ represents the target-driven loss. The weights α, β, and γ serve to balance these loss components.

#### 3.2.1. Content Loss

To enhance the overall visual quality and improve quantitative evaluation in the fusion model, a composite content loss mechanism is introduced. This loss mechanism consists of two main components: intensity loss $L_{\mathrm{int}}$ and texture loss $L_{\mathrm{tex}}$. The specific expression is as follows:

(2) $$L_{\mathrm{con}}=L_{\mathrm{int}}+\lambda L_{\mathrm{tex}}$$

In this equation, $L_{\mathrm{con}}$ represents the overall content loss, which is a weighted sum of the intensity loss $L_{\mathrm{int}}$ and the texture loss $L_{\mathrm{tex}}$. The weight λ is used to balance the contribution of the texture loss to the overall content loss.

$L_{\mathrm{int}}$ quantifies the pixel-level discrepancies between the synthesized and original images. Accordingly, the intensity loss for both infrared and visible spectra can be formulated as:

(3) $$L_{\mathrm{int}}=\frac{1}{HW}\left(\sum_{i=1}^{H}\sum_{j=1}^{W}∥I_{f}-I_{v}{∥}_{F}^{2}+\sum_{i=1}^{H}\sum_{j=1}^{W}{∥I_{f}-I_{r}∥}_{F}^{2}\right)$$

where ${∥\cdot ∥}_{F}$ denotes the Frobenius norm.$I_{f},I_{v},\;\mathrm{and}\;I_{r}$ represent the pixels of fused image, visible image, and infrared image, respectively. *H* and *W* represents the height and width of the image. The intensity loss focuses on preserving overall brightness and contrast, enhancing the visual quality of the fused image.

In addition, the incorporation of gradient loss imposes stricter network constraints, compelling the composite image to exhibit enhanced textural specificity and well-defined object boundaries. The gradient loss is formulated as:

(4) $$L_{\mathrm{tex}}=\frac{1}{HW}\left(\sum_{i=1}^{H}\sum_{j=1}^{W}∥|\nabla I_{f}|-|\nabla I_{v}{|∥}_{F}^{2}+\sum_{i=1}^{H}\sum_{j=1}^{W}∥|\nabla I_{f}|-|\nabla I_{r}{|∥}_{F}^{2}\right)$$

where ∇ denotes the gradient operator, and the Sobel operator is employed to calculate the gradient of the image.

#### 3.2.2. Detection Loss

The fused image is input into the YOLO detection network, and the detection loss is computed based on the predicted results and the ground truth. The returned detection loss comprises objectness loss, classification loss, and bounding box regression loss. The specific formulation for the detection-driven loss $L_{\det}$ is defined as follows:

(5) $$L_{\det}=\lambda_{\mathrm{loc}}I\left[y=1\right]L_{\mathrm{loc}}\left(b,\hat{b}\right)+\lambda_{\mathrm{cls}}I\left[y=1\right]L_{\mathrm{cls}}\left(p,\hat{p}\right)+\lambda_{\mathrm{obj}}L_{\mathrm{obj}}\left(o,\hat{o}\right)$$

where I[y=1] serves as the indicator function that is 1 when y=1 and 0 otherwise. $L_{\mathrm{cls}}\left(p,\hat{p}\right)$ denotes the classification loss and quantifies the discrepancy between the predicted class probabilities $\hat{p}$ and the ground truth *p*. Similarly, the objectness loss $L_{\mathrm{obj}}\left(o,\hat{o}\right)$ measures the difference between the predicted objectness $\hat{o}$ and the actual ground truth *o*. Both the classification loss and the objectness loss employ the cross-entropy loss function. $L_{\mathrm{loc}}\left(b,\hat{b}\right)$ denotes the localization loss between predicted bounding box $\hat{b}$ and the ground truth *b*, calculated using the CIOU loss function. The specific formulas are the same as in [49].

#### 3.2.3. Target Loss

In the composite image, the target object often attracts greater attention and thus holds more significance than other regions. Therefore, instead of applying a uniform treatment to the entire fused image, a target-specific loss is introduced. This loss utilizes a binary mask to indicate regions of interest based on the ground-truth bounding boxes.

The specific definition is as follows:

Let $B=\{\left(x_{c},y_{c},w,h\right)\}$ denote the set of ground-truth bounding boxes for the objects in the images, where $\left(x_{c},y_{c}\right)$ specifies the coordinates of the center of the bounding box, and *w* and *h* represent the width and height of the bounding box, respectively.

(6) $${\mathrm{Mask}}_{\mathrm{tar}}\left(i,j\right)=f\left(i,j;B\right)=\left\{\begin{array}{cc} 1 & \mathrm{if}\;(i,j)\;\mathrm{lies}\;\mathrm{within}\;\mathrm{bounding}\;\mathrm{box}\;\mathrm{in}\;B\\ 0 & \mathrm{otherwise} \end{array}\right.$$

(7) $$L_{\mathrm{tar}}=\frac{1}{HW}\sum_{i=1}^{H}\sum_{j=1}^{W}\left(∥{\mathrm{Mask}}_{\mathrm{tar}}⊙\left(I_{f}-\max\left(I_{v},I_{r}\right)\right){∥}_{1}+∥{\mathrm{Mask}}_{\mathrm{tar}}⊙(|\nabla I_{f}|-\max(|\nabla I_{v}|,|\nabla I_{r}{|))∥}_{1}\right)$$

where ⊙ denotes the Hadamard product. ${∥\cdot ∥}_{1}$ denotes the $\ell_{1}$ norm, and max(·) represents the maximum value of an element.

To emphasize the target and enhance its global contrast, the max function is used to preserve the highest pixel intensity and texture information from both infrared and visible images. This approach differs from traditional methods that typically extract intensity from infrared and texture from visible images. It is recognized that in complex real-world scenarios, visible images also contain brightness information influenced by lighting conditions, while infrared images have texture details stemming from varying thermal levels. Consequently, an equal approach is applied to both infrared and visible images.

### 3.3. Network Architecture

The architecture of the Local Texture Encoder (LTE) module is depicted in Figure 4. This module comprises two primary components: a Dense Connection Network and a set of three Gradient Feature Extraction Modules (Gblocks). Within each Gblock, a dual-branch feature extraction mechanism is introduced, incorporating gradient convolution and residual connections. The convolutional branch employs two 3 × 3 convolutional kernels, coupled with leaky ReLU as the activation function. In the gradient extraction branch, the Sober operator is used to extract gradient information along the *x*-axis and *y*-axis. To reconcile channel dimension discrepancies and bolster cross-channel feature integration, both branches terminate in a 1 × 1 convolution layer. Residual connections are then used to link the branches. By employing Gblocks, the LTE module excels at capturing fine-grained information. To further exploit the gradient-convolutional features across layers, a dense network is employed to connect the three Gblocks. This design enables each layer’s input to encompass the output of all preceding Gblocks, thereby achieving a comprehensive fusion of depth-specific and detail-oriented features.

Given the interdependence between the fusion model and the subsequent detection model, the architecture emphasizes the fusion model’s ability to extract global contextual information. The GST module, based on the architecture of Vision Transformer (ViT) [38], has been adapted to better suit the image fusion domain. The key improvements are as follows: Firstly, the original ViT operates only in the spatial dimension, whereas this module employs parallel Spatial ViT and Channel ViT, combining their outputs element-wise at the end. Secondly, unlike the original ViT which takes images directly as input, this model utilizes feature maps. This approach stems from the LTE module’s focus on image details, which in turn allows the GST module to focus on higher-level semantic information. Thirdly, the original ViT employs an MLP to revert vector groups to the original image size. The GST model, however, uses transposed convolution in place of fully connected layers, facilitating a gradual restoration of the image to its original size and better suiting image generation tasks.

The computational processes of the Spatial Transformer and Channel Transformer are illustrated in Figure 2 and Figure 3, respectively. In Spatial Transformer, *p* denotes the patch size, while *w* and *h* signify the number of image patches along the width and height dimensions, respectively. Furthermore, *E* represents the dimension of the embedding space. The input feature map is initially partitioned into a grid of p×p patches, which are subsequently flattened into one-dimensional vectors. These vectors undergo a linear transformation to project them into an *E*-dimensional embedding space. Then, these enriched vectors are then fed into the Transformer’s encoder layer for further processing. Finally, with transposed CNN, the vectors are reshaped to the same size as the original features.

In addition to spatial considerations, the architecture incorporates a Channel-based Visual Transformer to specifically capture inter-channel dependencies.The input feature map is segmented along the channel dimension into C’ groups, and these segments are then flattened into one-dimensional vectors. Following this, the vectors undergo a linear transformation to be projected into an *E*-dimensional embedding space. Subsequently, these vectors are input into the encoder layers of the Transformer for further processing. This facilitates a more comprehensive calculation of inter-channel relationships. Finally, using Multi-Layer Perceptron, the vectors are reshaped back to the dimensions of the original feature map. Given the absence of positional relevance in channel dimensions, positional embeddings are intentionally omitted. Collectively, the Spatial and Channel Transformers offer a comprehensive framework for relational mapping in image fusion tasks.

Through the cascade method, the features obtained by the LTE branch and the GST branch are integrated, and the combined data are sent to the image reconstruction module for feature fusion and final image generation. The reconstruction module consists of a sequence of three 3 × 3 convolutional layers followed by a single 1 × 1 convolutional layer. Each convolutional layer is succeeded by a normalization layer. Leaky ReLU serves as the activation function for the 3 × 3 convolutional layers, while Tanh is employed for the 1 × 1 convolutional layer.

## 4. Experiments

Section 4.1 details the experimental configurations and implementation specifics. Subsequently, Section 4.2 introduces the comparison methods and objective evaluation metrics. Comparative and generalization experiments are presented in Section 4.3 and Section 4.4, respectively, underscoring the advantages of TDDFusion. Section 4.5 delves into comparative experiments on target detection, showcasing the enhanced performance of the model in downstream detection tasks. Finally, Section 4.6 offers ablation experiments, emphasizing the functionality of different model components and affirming the rationality of our architecture.

### 4.1. Experimental Configuration and Implementation Details

The model was trained using the LLVIP dataset [25]. This dataset comprises 33,672 aligned images, equivalent to 16,836 pairs. The dataset was randomly split: 12,545 image pairs for the training set, 2742 pairs for the validation set, and 1549 pairs for the test set. Randomly selected sets of 42 pairs from the test set were employed to comprehensively verify the performance of the model. To further verify the effectiveness of the method, 37 pairs of images from TNO [26] and 42 pairs from RoadScene [27] were randomly chosen for generalization experiments. Additionally, 100 image pairs from both the LLVIP and Roadscene datasets were randomly selected and manually annotated to conduct object detection experiments.

To accommodate the requirements of the vision transformer in this study, all training images were uniformly resized to a resolution of 640×640 pixels, maintaining the original aspect ratio, and zero-padding was performed. Additionally, RGB images were transformed to the YCbCr color space, using only the Y channel for the image fusion process. After fusion, the images were converted back to the RGB color space. As a result of these image preprocessing steps, the size of the test images used in the testing phase could be inconsistent with the size of the training images.

The experiment adopted an alternating training approach between the fusion model and the detection model, as illustrated in Algorithm 1. The specific parameters were set as follows: M=3, *p*${}_{1}$ = 12,500, *p*${}_{2}$ = 15,600, $b_{1}=3$, and $b_{2}=8$. In addition, the hyper-parameters of the total loss were set as follows: α=1, β=0.5, γ=97. The hyper-parameter of the content loss was empirically set as λ=5. The Adam optimizer was employed with the following typical initialization parameters: α=0.001, $\beta_{1}=0.9$, $\beta_{2}=0.999$, and $\epsilon =10^{-8}$. YOLOv5s was utilized as the pre-trained model for detection with the default parameters. All experiments were conducted on a computer with an Intel(R) Core(TM) i7-12700KF CPU @ 3.60 GHz and an NVIDIA GeForce RTX 3090Ti GPU. The proposed deep model was implemented on the PyTorch platform.
**Algorithm 1** Training procedure1:**Input:** Infrared images $I_{r}$ and visible images $I_{v}$2:**Output:** Fused images $I_{f}$3:**for** *M* iterations **do**4:      **if** *M* == 0 **then**5:            **for** $p_{1}$ steps **do**6:                  Select $b_{1}$ infrared images $\left\{I_{r}^{1},I_{r}^{2},\cdots ,I_{r}^{b_{1}}\right\}$7:                  Select $b_{1}$ visible images $\left\{I_{v}^{1},I_{v}^{2},\cdots ,I_{v}^{b_{1}}\right\}$8:                  Calculate the content loss $L_{con}$ according to Equation (2)9:                  Update the parameters of the fusion sub-network $N_{F}$ by Adam Optimizer: $\nabla_{N_{F}}\left(L_{con}\right)$10:           **end for**11:      **else**12:           **for** $p_{1}$ steps **do**13:                Select $b_{1}$ infrared images $\left\{I_{r}^{1},I_{r}^{2},\cdots ,I_{r}^{b_{1}}\right\}$14:                Select $b_{1}$ visible images $\left\{I_{v}^{1},I_{v}^{2},\cdots ,I_{v}^{b_{1}}\right\}$15:                Calculate the total loss $L_{total}$ according to Equation (1)16:                Update the parameters of the fusion sub-network $N_{F}$ by Adam Optimizer: $\nabla_{N_{F}}\left(L_{Total}\right)$17:           **end for**18:      **end if**        Generate fusion images from infrared and visible images in the training set;19:      **for** $p_{2}$ steps **do**20:           Select $b_{2}$ fusion images $\left\{I_{f}^{1},I_{f}^{2},\cdots ,I_{f}^{b_{2}}\right\}$21:           Update the parameters of the YOLOv5 sub-network $N_{Y}$ by SGD Optimizer: $\nabla_{N_{Y}}\left(L\left(N_{Y}\right)\right)$22:      **end for**23:**end for**

### 4.2. Comparison Methods and Evaluation Indicators

To fully demonstrate the effectiveness of the method, it was compared with seven state-of-the-art methods in generalization experiments, including two representative traditional methods, namely GTF [50], ADF [51], and five deep learning-based methods, namely DenseFuse [52], FusionGAN [19], RFN-Nest [30], stdfusion [23], and TarDAL [48]. In the experiment of detection performance and comparative experiment, comparative tests were conducted with SeAFusion [5] in addition to the aforementioned seven methods. The code for all these methods is publicly accessible. The optional parameters were configured as detailed in their respective papers.

For quantitative evaluation, six metrics were selected to objectively assess the fusion performance, including average gradient (AG), entropy (EN) [53], mutual information [54], gradient-based fusion performance $Q_{abf}$, standard deviation (SD), and visual information fidelity (VIF) [55]. AG is used to determine the amount of detail or sharpness present in an image. EN measures of the amount of information or complexity in the image. MI quantifies the amount of information derived from the source images in the fused image. The $Q_{abf}$ metric quantifies the transfer of edge information from the source images to the resultant fused image. SD provides insight into the contrast and distribution variability of fused images from a statistical standpoint. VIF evaluates the similarity between source images and the fusion image from the perspective of the human visual system. Additionally, higher values of AG, EN, MI, $Q_{abf}$, SD, and VIF suggest superior fusion results.

### 4.3. Comparative Experiment

Comparative experiments were conducted on the LLVIP dataset, assessing both qualitatively and quantitatively against seven other methodologies, to comprehensively evaluate the fusion performance of the approach.

#### 4.3.1. Qualitative Results

**Figure 5:**
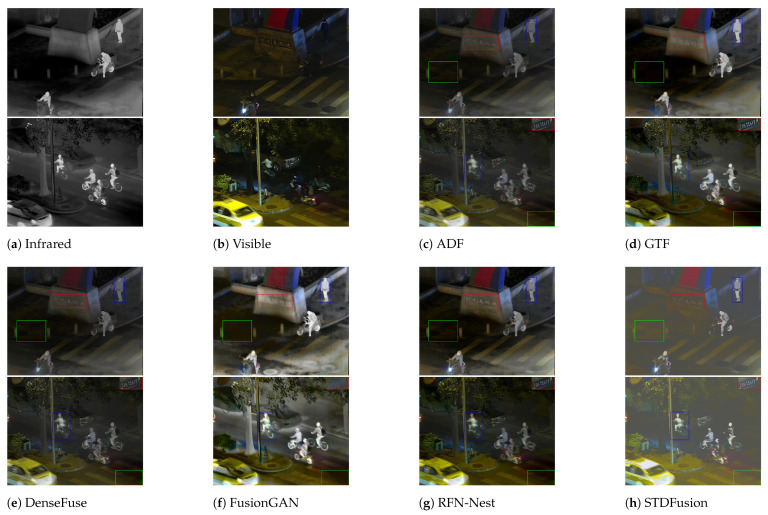

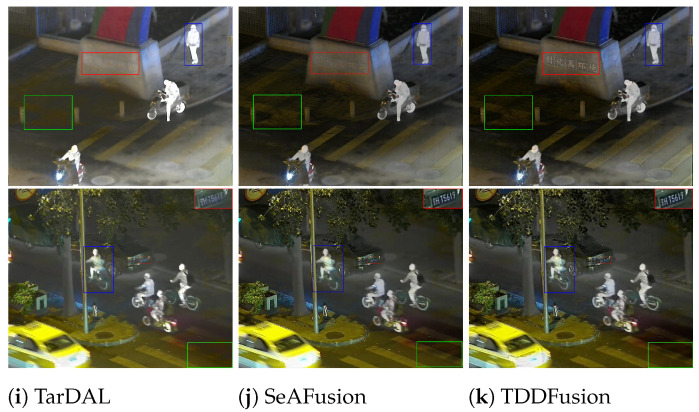
Qualitative analysis of TDDFusion with seven methods on 010014 (**top**) and 080077 (**bottom**) images from the LLVIP dataset.

Qualitative comparisons of different algorithms on the LLVIP dataset are shown in Figure 5. In comparison with the other seven methods, the proposed approach, TDDFusion, exhibits superior performance in three distinct aspects. First, the method strikes a balance between contrast and detail preservation of the target. It not only effectively retains intensity information but also maintains certain textural details of the targets. Second, the proposed approach can preserve the rich local details in the images, showcasing a higher clarity in the local textual elements. Lastly, the method adeptly retains the contrast information provided by illumination in the visible light images, thus better preserving environmental context.

In the first set of pictures in Figure 5, first of all, compare the pedestrian target in the blue box in the upper left corner. The five methods of GTF, FusionGAN, TarDAL, SeAfusion, and TDDFusion can effectively emphasize the human figure, among which TarDAL, SeAfusion, and TDDFusion have clearer outlines. While TarDAl has higher contrast, it falls short in highlighting texture details, since it relies more on infrared intensity information. Notably, TDDFusion provides a clearer representation that the pedestrian is wearing a mask on their face. Secondly, the clarity of the text within the red box reflects the capability of TDDFusion to preserve local detail information. Except for FusionGAN and TarDAL, the textual content is discernible in the outputs of all other methods. Yet, in terms of clarity and contrast of the text, RFN-Nest and TDDFusion hold an advantage. Lastly, the road environment information displayed within the green box on the left side of the image can be inferred from the subtle light contrast in the visible light image. Among the fusion results, only STDFusion, SeAfusion, and TDDFusion manage to retain this road environment information.

In the second set of images in Figure 5, we magnified the license plate within the red box and positioned it in the top right corner of the image. Apart from FusionGAN and TarDAL, the letters and numbers on the license plate are discernible in the outputs of all other methods. Specifically, GTF, STDFusion, SeAfusion, and TDDFusion effectively reveal the Chinese characters on the plate. In the target of pedestrian indicated by the blue box, TarDAL, SeAfusion and TDDFusion still exhibit the best contrast and contour definition. In the green box located at the bottom right corner of the visible light image, faint zebra crossing lines under low lighting conditions are displayed. Among the fusion results, only STDFusion, SeAfusion, and TDDFusion manage to adequately preserve this information.

#### 4.3.2. Quantitative Result

**Figure 6:**
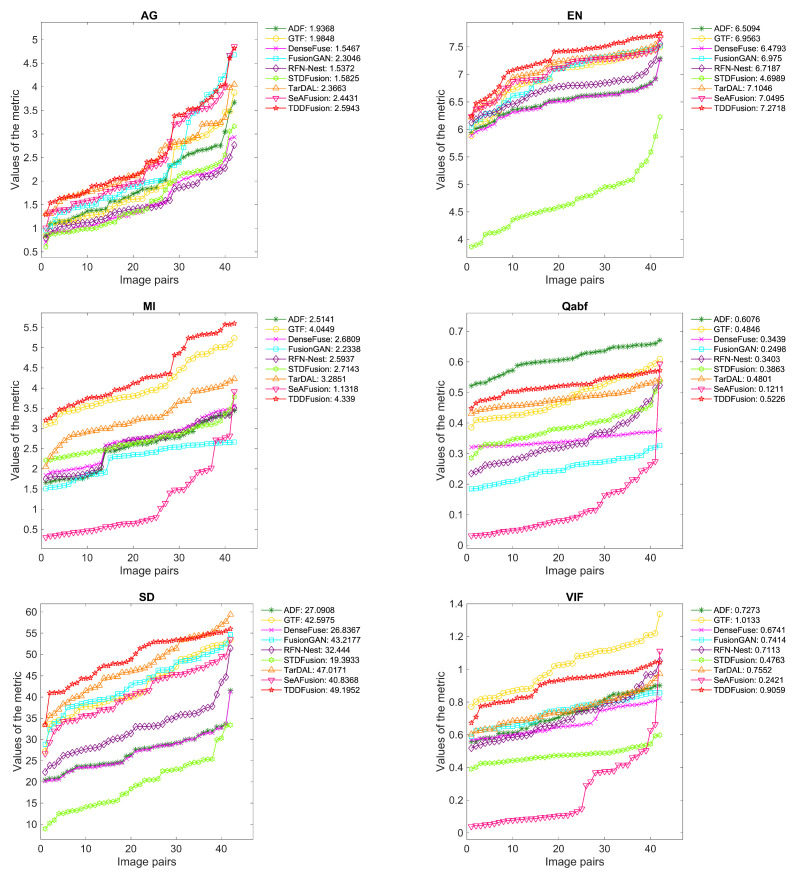
Performance graph of different fusion methods on LLVIP dataset. The *x*-axis represents image pairs and the *y*-axis represents the values of the metric. For ease of observation, the evaluation values for each method were arranged in ascending order. The average value is shown in the legend.

A selection of 42 image pairs from the LLVIP dataset were used for quantitative evaluation. Figure 6 displays the quantitative results for six complementary metrics. Notably, the proposed method demonstrated significant superiority in the AG, EN, MI, and SD metrics. The top-performing AG and SD indicate that TDDFusion possesses the richest detail information and high contrast. The optimal EN and MI scores suggest that TDDFusion transferred the maximum amount of information from the source images to the fused image, retaining the highest information content. Although the method was not the best in the $Q_{abf}$ and VIF metrics, it produced the second-best fusion results. This underscores the capability of the method in generating fused images that align with human visual characteristics.

### 4.4. Generalization Experiment

It is widely acknowledged that the generalization capability is crucial for deep learning methodologies. To this end, experiments on the TNO and RoadScene dataset were conducted to showcase the adaptability of the proposed method. Notably, the fusion model, initially trained on the LLVIP dataset, was directly evaluated on the TNO and RoadScene dataset without further tuning.

#### 4.4.1. Results of TNO

**Figure 7:**
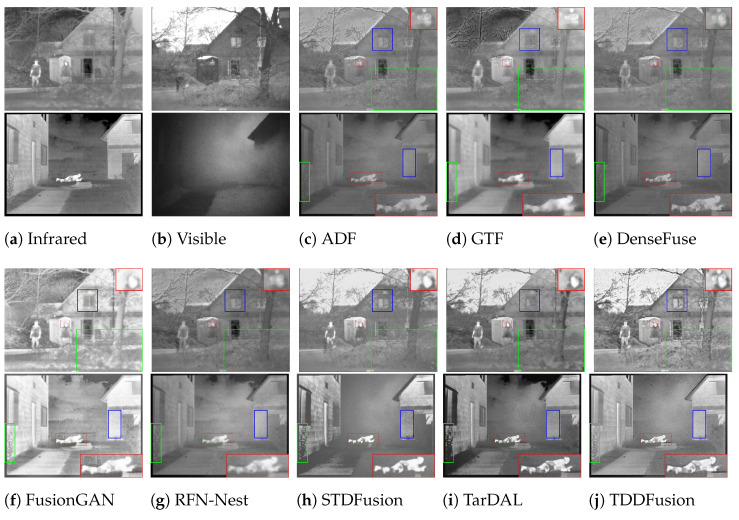
Qualitative analysis of TDDFusion with seven methods on two representative images from the TNO dataset.

Qualitative comparisons of different algorithms on the TNO dataset are presented in Figure 7. In the first set of images, the portrait within the red box is magnified and placed in the top right corner of the image. Only STDFusion, TarDAL, and TDDFusion offer a relatively clear representation of the subject. Notably, TDDFusion is the sole method capable of preserving subtle details like a strand of floating hair, highlighting its proficiency in emphasizing the main target while retaining intricate textures. Furthermore, the areas marked by the blue and green boxes represent the global background environment. It is evident that only ADF, STDFusion, and TDDFusion manage to clearly retain extensive background details. Among these three, TDDFusion delivers superior contrast and has a more visually appealing result.

In the second set of images featuring a smoky scenario, the target is completely obscured by smoke in the visible light image. Within the fused images, the subject in the red box is magnified and placed in the bottom right corner. While all methods reveal the subject, only TarDAL and TDDFusion manage to retain the subject clearly. The other methods suffer from issues like low contrast, blurred contours, or ghosting artifacts. Regarding the environmental information in the areas marked by the blue and green boxes, TDDFusion stands out among the methods. It notably preserves both the sharp edges of the leaves and the wall brick textures, resulting in a more visually pleasing effect.

**Figure 8:**
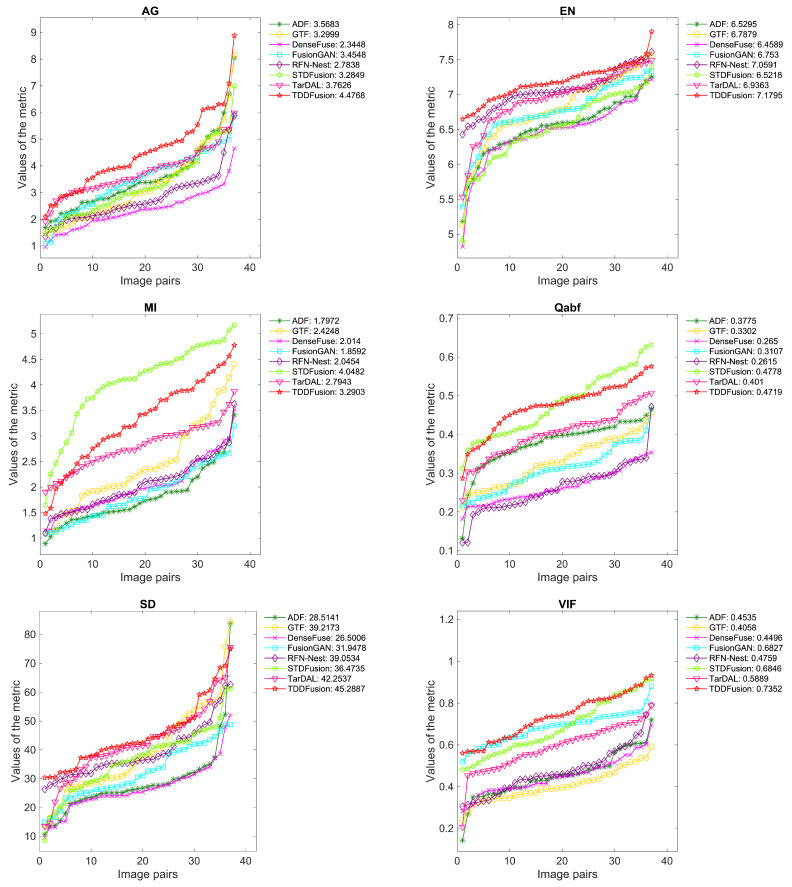
Performance graph of different fusion methods on TNO dataset. The *x*-axis represents image pairs and the *y*-axis represents the values of the metric. For ease of observation, the evaluation values for each method were arranged in ascending order. The average value is shown in the legend.

Quantitative Comparison: A selection of 37 pairs of images from the TNO dataset was used for a quantitative evaluation. The objective assessment results are shown in Figure 8. Similar to the metrics on the LLVIP dataset, TDDFusion achieved the best overall performance, demonstrating significant advantages in the AG, EN, SD, and VIF metrics on the TNO dataset. Such results imply that the proposed method can retain more texture detail and produce visually pleasing fusion outcomes. The $Q_{abf}$ metric is only slightly behind the best result. As for the MI metric, the proposed approach achieved a near-optimal performance. Experimental results validate the consistent reliability of the algorithm on the TNO dataset.

#### 4.4.2. Results of RoadScene

**Figure 9:**
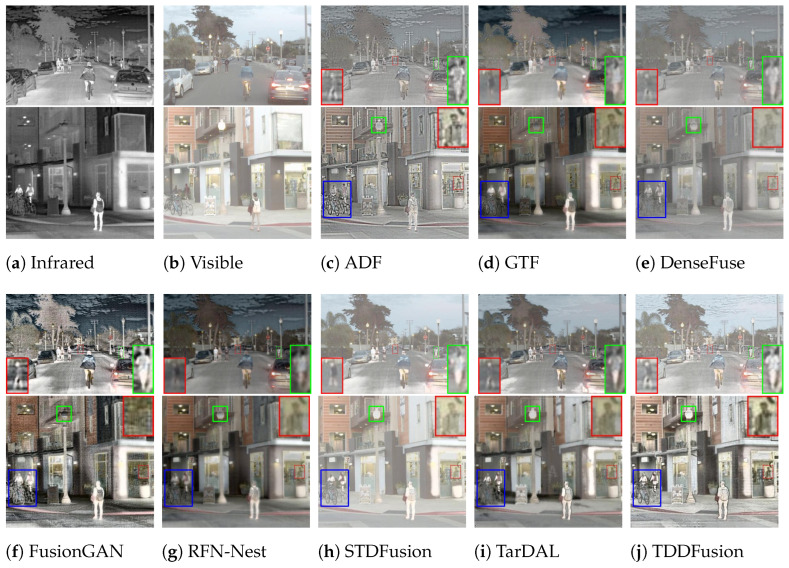
Qualitative analysis of TDDFusion with seven methods on FLIR-06832 (**top**) and FLIR-08835 (**bottom**) images from the RoadScene dataset.

Qualitative comparisons of different algorithms on the Roadscene dataset are illustrated in Figure 9. In the first set of images, the pedestrian highlighted in the red box is magnified and placed in the bottom right corner. Notably, GTF, DenseFuse, and RFN-Nest fail to maintain the intensity distribution of the prominent target region, causing the distant pedestrian to blend with the background. Among the remaining methods, only FusionGAN and TDDFusion exhibit superior contrast and retain a discernible target outline. The pedestrian in the green box is magnified and displayed in the bottom right corner. From the magnified images, it can be observed that DenseFuse, FusionGAN, TarDAL, and TDDFusion preserve the intensity distribution of the significant human region. However, only TDDFusion offers enhanced contrast and aligns closely with human visual perception.

In the second set of images, the target individual located indoors, highlighted in the red box, is magnified and positioned in the upper right corner of the image. Both DenseFuse and FusionGAN struggle to maintain the intensity distribution of the significant target area. However, only ADF and TDDFusion can clearly retain the figure. Other methods, due to insufficient information integration, result in the loss of texture from the visible image. The proposed approach, fortified by the target loss and GST module, enhances the subject focus and its global association, achieving a better integration of complementary and shared features from the source images in the target region.

**Figure 10:**
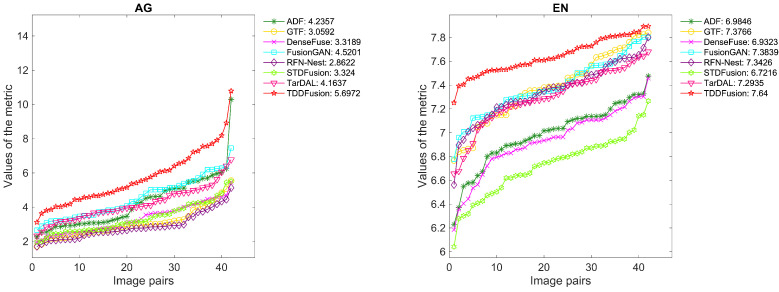

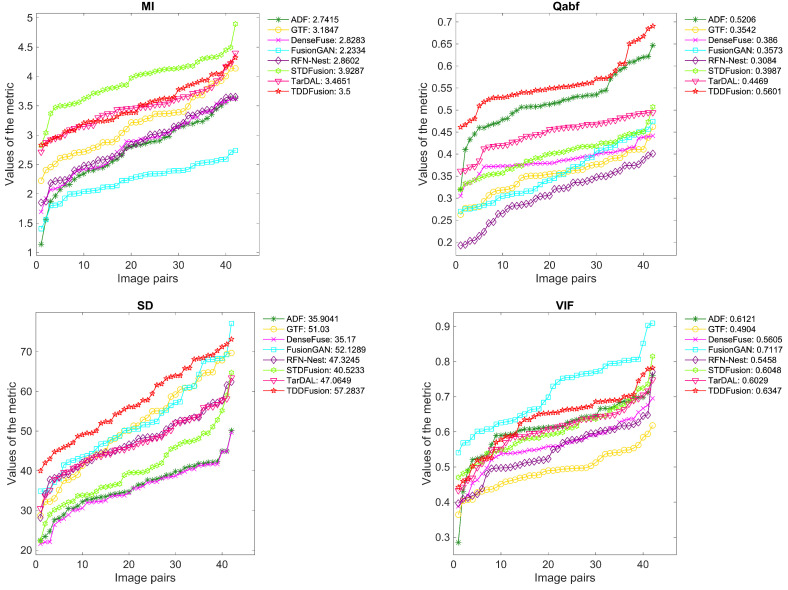
Performance graph of different fusion methods on Roadscene dataset. The *x*-axis represents image pairs and the *y*-axis represents the values of the metric. For ease of observation, the evaluation values for each method were arranged in ascending order. The average value is shown in the legend.

Quantitative Comparison: A selection of 42 image pairs from the Roadscene dataset was used for quantitative evaluation. Figure 10 depicts the quantitative results across six complementary metrics. TDDFusion demonstrated exceptional performance on all the metrics. The superior EN indicates that the fused images encompass the maximal information, while the highest $Q_{abf}$ and AG affirm that the fusion approach retains the most edge details. Additionally, the top-ranking SD underscores that the fused images present favorable contrast. While MI and VIF are slightly behind the best outcomes, the disparity is negligible. Overall, both qualitative and quantitative experiments underscore the commendable generalization capacity of TDDFusion.

### 4.5. Detection Performance

To further evaluate the performance of different methods on the detection task, a test set comprising 100 image pairs was randomly selected. Of these, 50 pairs originate from the LLVIP dataset, while the other 50 pairs are sourced from the Roadscene dataset. These 100 pairs encompass both daytime and nighttime settings and include the majority of urban scenarios. Manual annotations for humans and cars were performed in these images. To ensure a fair comparison, detection on all fused images was carried out using a pre-trained YOLOv5 model. Quantitative evaluations were undertaken using the mean Average Precision (mAP). The mAP@0.5 represents the mAP value at an IoU threshold of 0.5, while mAP@[0.5:0.95] denotes the average mAP across IoU thresholds ranging from 0.5 to 0.95 with a step size of 0.05. The mAP score lies between 0 and 1, with values closer to 1 indicating better detection outcomes by the model.

As depicted in Table 1, IR images with prominently highlighted thermal structures of targets significantly aid in human detection, whereas VI images offer rich semantic information about vehicles. For composite category detection, the performance of various fusion models generally surpasses that of single-source detection. Among the array of fusion methods, TDDFusion particularly excels in human detection. This advantage is attributed to the target loss specifically designed for pedestrian objectives during training, substantially enhancing the image quality within target areas. Experimental outcomes indicate that the proposed model consistently achieves optimal comprehensive detection capabilities across various IoU thresholds.

**Figure 11:**
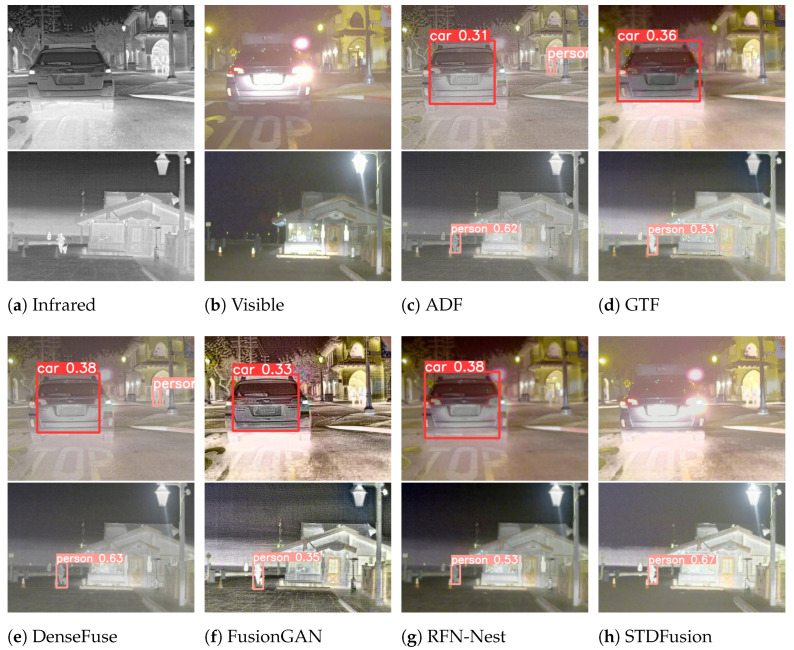

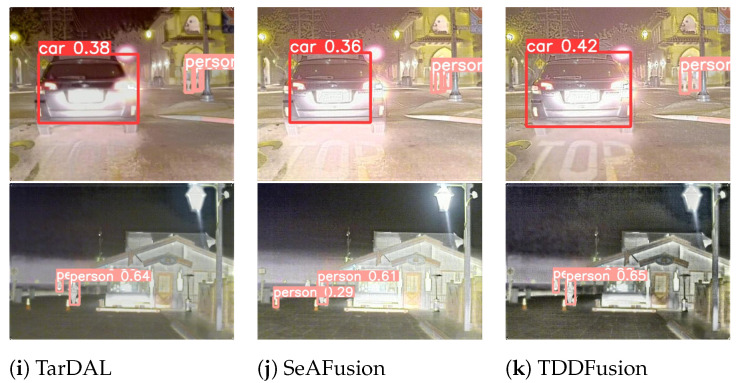
Qualitative analysis of TDDFusion with seven methods on images of FLIR-09652 (**top**) and FLIR-05914 (**bottom**) from the RoadScene dataset.

Simultaneously, a visual presentation of the detection results is offered. As illustrated in Figure 11, in the first set of images, both ADF and DenseFuse detect one of the two pedestrians in the top right corner, while only TarDAL, SeAFusion, and TDDFusion successfully identify both. The remaining algorithms entirely miss the pedestrians in that area. As for vehicle detection, only the fused image from STDFusion fails in the detection task. In the second set of images, only the fused images from TarDAL and TDDFusion can detect the distant pedestrian. Additionally, SeAFusion has one false detection. Although both TarDAL and TDDFusion successfully identify all vehicles and pedestrians in these two sets, TDDFusion’s confidence level still surpasses that of TarDAL. This conclusively illustrates that TDDFusion can offer abundant semantic information, showcasing superior performance in detection tasks.

### 4.6. Ablation Study

To rigorously ascertain the contributions of individual modules within the framework, systematic ablation studies were executed. The impact of these modules was quantified through metrics such as AG, EN, MI, $Q_{abf}$, SD, and VIF, with results presented in Table 2. The evaluation also extended to object detection tasks, utilizing the annotated test dataset mentioned in Section 4.5. Detection performance was assessed using metrics mAP@0.5 and mAP@[0.5:0.95], with results detailed in Table 3.

In the proposed model of this study, the detection loss and target loss play a crucial role during the training process. According to Equation (1), β and γ, as hyperparameters, control the weight of each part of the loss. To ascertain the optimal settings for the hyperparameters beta and gamma in the deep learning model, a series of ablation experiments were conducted. These experiments involved varying the values of β and γ to understand their impact on the model’s performance. Specifically, β was tested at 0, 5, and 50, corresponding to models M1, M2, and M3, respectively. γ was tested at 0, 1, and 10, corresponding to models M4, M5, and M6. Moreover, at the architectural level, the GST module is instrumental in extracting intricate semantic information. To further probe its significance, an additional fusion model, excluding the GST module and denoted as M7, was trained.

Adjusting the value of β allows for modulation of the detection loss weight. As indicated in Table 2, setting β to 0 results in minor declines in all metrics. Notably, VIF exhibits a slight increase when detection loss is excluded. Furthermore, as β increases, all metrics significantly deteriorate, suggesting a delicate equilibrium between these two aspects in the model’s architecture. Eliminating the target loss leads to a modest decline in all metrics. Additionally, minor variations in the γ value have minimal impact on the metrics. When the GST module is removed, both EN and $Q_{abf}$ metrics show significant decreases, highlighting the GST module’s pivotal role in augmenting the overall information content and high-frequency details of the images.

As demonstrated in Table 3, eliminating the detection loss significantly diminishes detection accuracy at various IOU thresholds. However, excessively high values of β lead to even lower scores. This decrease is attributed to an inappropriate value of β, which affects the image quality, thereby reducing detection rates. The omission of the target loss, or setting γ to a very low value, substantially affects detection accuracy, especially for human subjects. Similarly, the removal of the GST module significantly affects detection accuracy, suggesting that the GST module plays a crucial role in extracting global information and is essential for improving the performance in subsequent detection tasks.

In summary, the results presented in Table 2 and Table 3 underscore the efficacy and cogency of the devised modules and the holistic architectural design of the network.

## 5. Conclusions

Within this paper, the TDDFusion framework, a dual-branch fusion system, is proposed. This framework successfully bridges the gap between pixel-level image fusion and downstream object detection performance. It integrates an object detection scheme into the fusion process, employing backpropagation of semantic loss for a task-driven approach. It features a dual-branch architecture with the GST branch for extracting global semantic information and the LTE branch for capturing local textures and contrasts. A novel loss function utilizing target positional information enhances visual contrast and detail for focused objects. Tests on three public datasets have shown TDDFusion’s effectiveness in maintaining global and local image details, balancing pixel intensity, and preserving target textures. Crucially, it demonstrates superior performance in subsequent object detection tasks, highlighting its practical advantages in task-oriented applications.

## Figures and Tables

**Table 1:** Object detection performance (measured as mAP) of source images and the fused images of different fusion methods. Within the assessment, the top-performing method is emphasized in red, while the second-best is denoted in blue.

	mAP@0.5			mAP@[0.5:0.95]		
	**Person**	**Car**	**All**	**Person**	**Car**	**All**
IR	0.7793	0.5076	0.6605	0.4375	0.2323	0.3537
VI	0.5293	0.7435	0.6172	0.2646	0.3689	0.3075
ADF	0.7427	0.7236	0.7355	0.3935	0.3418	0.3713
GTF	0.7639	0.7118	0.7297	0.4139	0.3359	0.3472
DenseFuse	0.7421	0.7419	0.7420	0.4005	0.3793	0.3721
FusionGAN	0.7469	0.7223	0.7298	0.4045	0.3515	0.3649
RFN-Nest	0.7616	0.7194	0.7368	0.4108	0.3422	0.3680
STDFusion	0.7745	0.7265	0.7547	0.4125	0.3779	0.3791
TarDAL	0.7821	0.7638	0.7712	0.4201	0.3721	0.3826
SeAFusion	0.7681	0.7305	0.7403	0.4022	0.3716	0.3692
TDDFusion	0.8026	0.7621	0.7863	0.4306	0.3775	0.4027

**Table 2:** Quantitative results of six indices under ablation experiments on LLVIP test set of 42 image pairs. Within the assessment, the top-performing method is emphasized in red, while the second best is denoted in blue.

	AG	EN	MI	Qabf	SD	VIF
**M1**	2.4336	7.0705	3.826	0.5203	47.1083	1.0175
**M2**	2.3059	6.8137	2.6172	0.5203	47.1083	0.7218
**M3**	2.3059	6.8137	2.6172	0.3235	41.3274	0.6130
**M4**	2.5297	7.2501	4.287	0.5221	48.9597	0.9021
**M5**	2.5299	7.2479	4.293	0.5214	48.9503	0.9027
**M6**	2.5360	7.2569	4.291	0.5223	48.9528	0.9033
**M7**	2.1792	6.7932	3.475	0.4375	46.2759	0.7559
**Ours**	2.5943	7.2718	4.3390	0.5226	49.1952	0.9059

**Table 3:** Object detection performance (measured as mAP) of different fusion methods under ablation experiments. Within the assessment, the top-performing method is emphasized in red, while the second best is denoted in blue.

	mAP@50			mAP@[0.5:0.95]		
	**Person**	**Car**	**All**	**Person**	**Car**	**All**
M1	0.7625	0.7526	0.7569	0.4127	0.3761	0.3794
M2	0.8013	0.7461	0.7401	0.4152	0.3694	0.3680
M3	0.7472	0.7197	0.7304	0.4046	0.3517	0.3657
M4	0.7718	0.7594	0.7628	0.4139	0.3726	0.3831
M5	0.7716	0.7593	0.7627	0.4139	0.3726	0.3832
M6	0.7735	0.7601	0.7701	0.4196	0.3694	0.3835
M7	0.7723	0.7318	0.7591	0.4203	0.3689	0.3729
Ours	0.8026	0.7621	0.7863	0.4306	0.3775	0.4027

## Data Availability

The authors confirm that the data supporting the findings of this study are available within the article.
